# Supplementary material for: The Spatial Distribution of Crimean–Congo Haemorrhagic Fever and Its Potential Vectors in Europe and Beyond
Source: Insects. 2023 Sep 17;14(9):771. doi: 10.3390/insects14090771 (PMC10532370; doi:10.3390/insects14090771)

Supplementary Information

Supplementary Table S1: Vector habitat suitability conditions

Variable	*H. marginatum*	*H. lusitanicum*	Dataset
Forest/woodland > 40%	No	Yes	Corine/ESA CCI
Woodland or Forest 15 - 40%	Yes	Yes	Corine/ESA CCI
Shrubland/Grassland	Yes	Yes	Corine/ESA CCI
Cropland	Yes	Yes	Corine/ESA CCI
Sparse Vegetation	Yes	No	Corine/ESA CCI
Minimum Elevation	< 2000m	< 2000m	GMTED (see URL below)
Max temp	< 35 ^{o}C	< 40 ^{o}C	Worldclim (see URL below)
Summer minimum Relative Humidity		>10%, < 65%	Derived from MODIS LST (see URL below)
Cumulative temperature above 15°C, April to August	>800, plus 50km buffer		Worldclim (see URL below)
Cumulative minimum temperature above 0 ^{o}C, October and November		>400	Worldclim (see URL below)
Mean Annual Vapour Pressure Deficit	< 15%		ECWMF (see URL below)

Corine Land Cover 2018 https://land.copernicus.eu/pan-european/corine-land-cover,

ESA CCI land Cover http://maps.elie.ucl.ac.be/CCI/viewer/download.php

GMTED https://topotools.cr.usgs.gov/gmted_viewer/gmted2010_global_grids.php

Modis Land Surface Temperature https://modis.gsfc.nasa.gov/data/dataprod/mod11.php

Worldclim: https://www.worldclim.org/data/index.html

ECMWF: https://www.ecmwf.int/en/forecasts/datasets/reanalysis-datasets/era5

Supplementary Table S2: Covariates offered to modelling procedures

1 VCC1103A0: Middle infra-red mean<br>2 VCC1103A1: Middle infra-red amplitude 1<br>3 VCC1103A2: Middle infra-red amplitude 2<br>4 VCC1103A3: Middle infra-red amplitude 3<br>5 VCC1103MN: Middle infra-red minimum<br>6 VCC1103MX: Middle infra-red maximum<br>7 VCC1103P1: Middle infra-red phase 1<br>8 VCC1103P2: Middle infra-red phase 2<br>9 VCC1103P3: Middle infra-red phase 3<br>10 VCC1103VR: Middle infra-red variance<br>11 VCC1107A0: Daytime LST mean<br>12 VCC1107A1: Daytime LST amplitude 1<br>13 VCC1107A2: Daytime LST amplitude 2<br>14 VCC1107A3: Daytime LST amplitude 3<br>15 VCC1107MN: Daytime LST minimum<br>16 VCC1107MX: Daytime LST maximum<br>17 VCC1107P1: Daytime LST phase 1<br>18 VCC1107P2: Daytime LST phase 2<br>19 VCC1107P3: Daytime LST phase 3<br>20 VCC1107VR: Daytime LST variance<br>21 VCC1108A0: Nighttime LST mean<br>22 VCC1108A1: Nighttime LST amplitude 1<br>23 VCC1108A2: Nighttime LST amplitude 2<br>24 VCC1108A3: Nighttime LST amplitude 3<br>25 VCC1108MN: Nighttime LST minimum<br>26 VCC1108MX: Nighttime LST maximum<br>27 VCC1108P1: Nighttime LST phase 1<br>28 VCC1108P2: Nighttime LST phase 2<br>29 VCC1108P3: Nighttime LST phase 3<br>30 VCC1108VR: Nighttime LST variance<br>31 VCC1114A0: NDVI mean<br>32 VCC1114A1: NDVI amplitude 1<br>33 VCC1114A2: NDVI amplitude 2<br>34 VCC1114A3: NDVI amplitude 3<br>35 VCC1114MN: NDVI minimum<br>36 VCC1114MX: NDVI maximum<br>37 VCC1114P1: NDVI phase 1<br>38 VCC1114P2: NDVI phase 2<br>39 VCC1114P3: NDVI phase 3<br>40 VCC1114VR: NDVI variance<br>41 VCC1115A0: EVI mean<br>42 VCC1115A1: EVI amplitude 1	43 VCC1115A2: EVI amplitude 2<br>44 VCC1115A3: EVI amplitude 3<br>45 VCC1115MN: EVI minimum<br>46 VCC1115MX: EVI maximum<br>47 VCC1115P1: EVI phase 1<br>48 VCC1115P2: EVI phase 2<br>49 VCC1115P3: EVI phase 3<br>50 VCC1115VR: EVI variance<br>51 VCMI30GRDP1K DEM (Elevation) +1000<br>52 VC1920A0: ERA5 Precipitation mean<br>53 VC1920A1: ERA5 Precipitation amplitude 1<br>54 VC1920A2: ERA5 Precipitation amplitude 2<br>55 VC1920A3: ERA5 Precipitation amplitude 3<br>56 VC1920MN: ERA5 Precipitation minimum<br>57 VC1920MX: ERA5 Precipitation maximum<br>58 VC1920P1: ERA5 Precipitation phase 1<br>59 VC1920P2: ERA5 Precipitation phase 2<br>60 VC1920P3: ERA5 Precipitation phase 3<br>61 VC1920VR: ERA5 Precipitation variance<br>62 VCWPOPPPP: Worldpop Human Population density 2020<br>63 VCV59EL500: GNTED Elevation + 500<br>64 VCEELCBARE: consensus % bare ground<br>65 VCEELCDCBD3: consensus % deciduous broadleaved forest<br>66 VCEELCEVGBD2: consensus % evergreen broadleaved forest<br>67 VCEELCEVGND1: consensus % evergreen needleleaved forest<br>68 VCEELCFLOOD8: consensus % flooded<br>69 VCEELCHVCB: consensus % herbaceous cover<br>70 VCEELCMANAG7: consensus % managed land<br>71 VCEELCOTHTR4: consensus % other land cover<br>72 VCEELCSHRUB5: consensus % shrub cover<br>73 VCEELCURB9: consensus % urban<br>74 VCEELCWATER12: consensus % water<br>75 VC82094A0: Relative Humidity mean<br>76 VC82094A1: Relative Humidity amplitude 1<br>77 VC82094A2: Relative Humidity amplitude 2<br>78 VC82094A3: Relative Humidity amplitude 3<br>79 VC82094MN: Relative Humidity minimum<br>80 VC82094MX: Relative Humidity maximum<br>81 VC82094P1: Relative Humidity phase 1<br>82 VC82094P2: Relative Humidity phase 2<br>83 VC82094P3: Relative Humidity phase 3<br>84VC82094VR: Relative Humidity variance

LST = Land Surface Temperature. NDVI Normalised Difference vegetation Index; EVI Enhanced Vegetation Index. DEM Digital Elevation. All files starting with VCC11, VC19 and VC82 are Fourier processed MODIS Satellite Imagery produced by the Environmental Research Group Oxford according to the methods set out in Scharlemann *et. al.* (2008) [24].

The Relative Humidity layers were produced as described in n Kraemer *et. al.* (2019)[38] and then Fourier Processed as described above.

The Elevation layer was extracted from the GMTED datasets (https://topotools.cr.usgs.gov/gmted_viewer/gmted2010_global_grids.php) and the negative values removed by adding 1000.

Population layers derived from layers produced by worldpop (https:// www.worldpop.org/datacatalog/)

All Files with VCEELC in file name were derived from the Earthenv consensus land cover data product (https://www.earthenv.org/landcover)

All layers extracted and standardised by ERGO for MOOD Horizon 2020 project N° 874850 (https://mood-h202.eu)

Supplementary Figure S1:VectorNet data locations

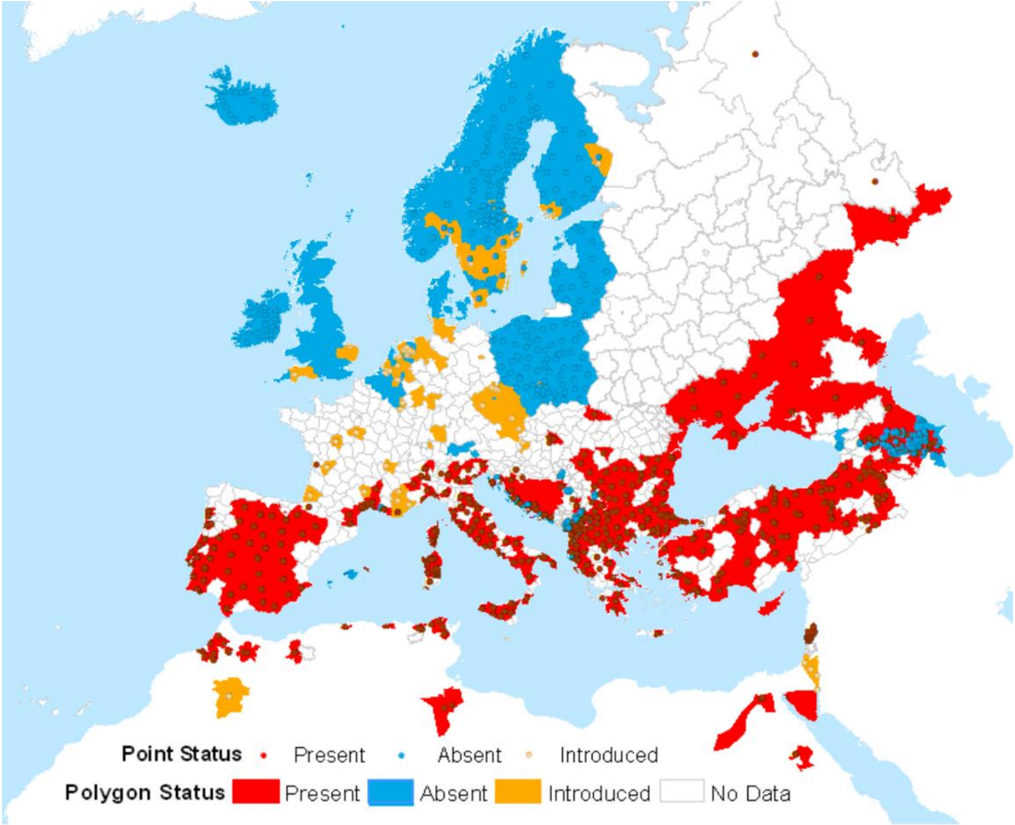

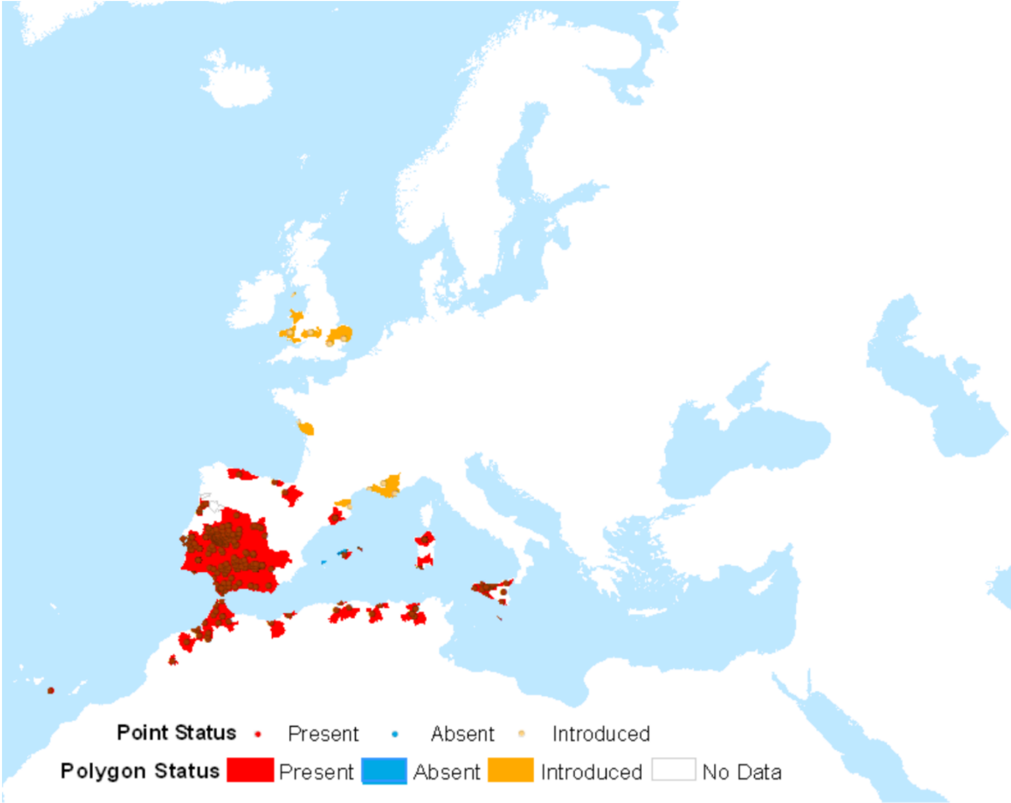

Top *H. marginatum*, Bottom *H. lusitanicum.*

Supplementary Figure S2: Habitat suitability for each vector.

Top *H. marginatum*, Bottom *H. lusitanicum.* Unsuitable = Green, Suitable=white

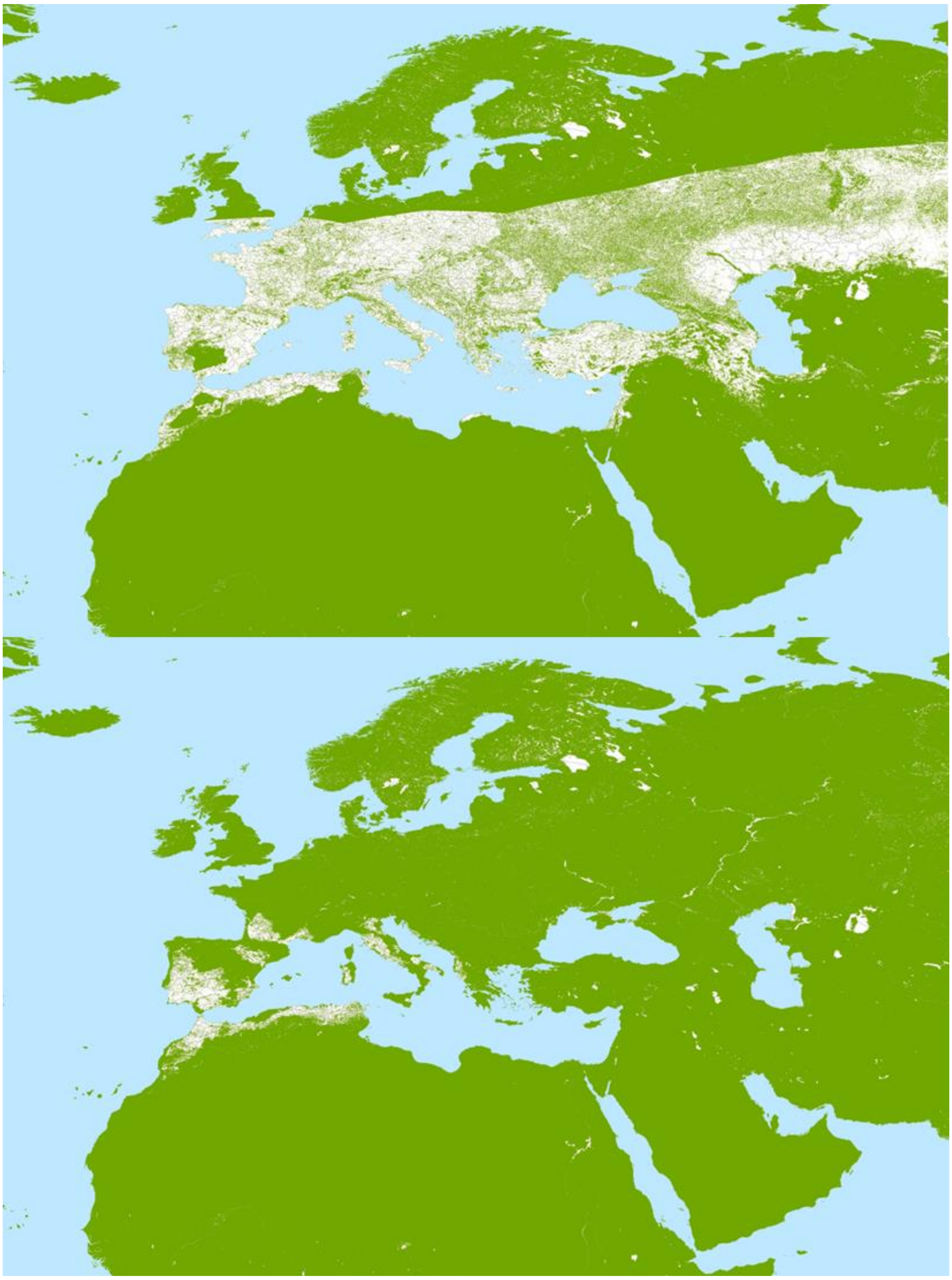

Supplementary Figure S3: Full extent of CCHF pseudo-absence and occurrence locations

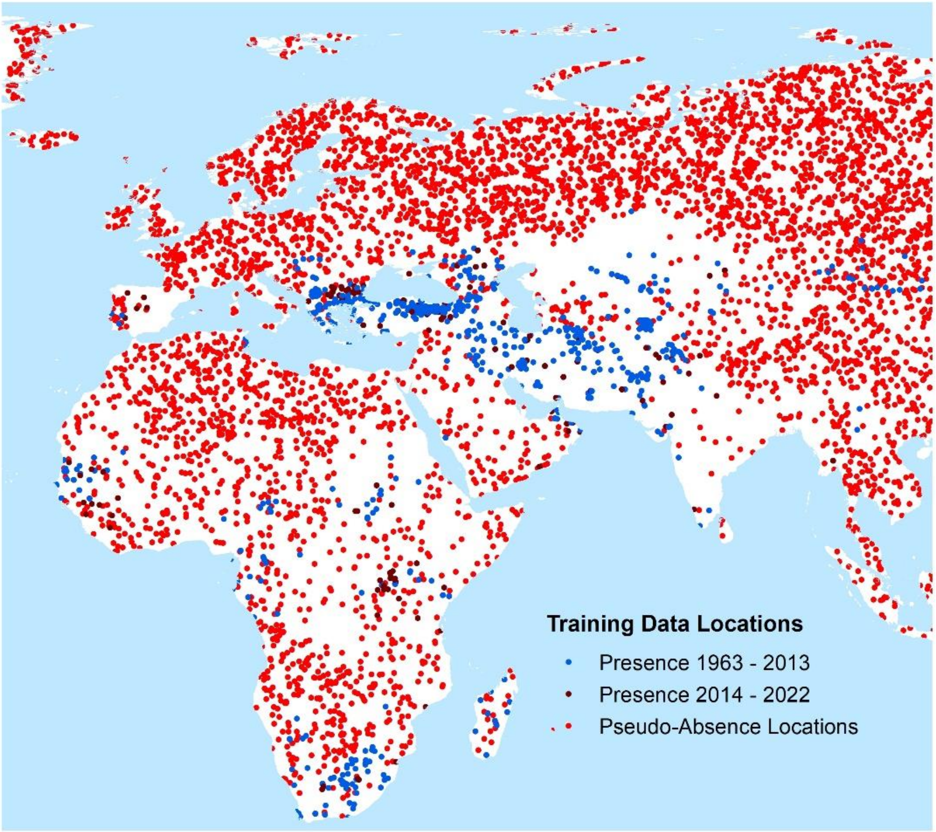

Supplementary Figure S4: Frequency histograms of CCHF human occurrence locations by year of published report. *Histograms by year of (A) point occurrence locations; (B) administrative area (polygon) occurrence locations*

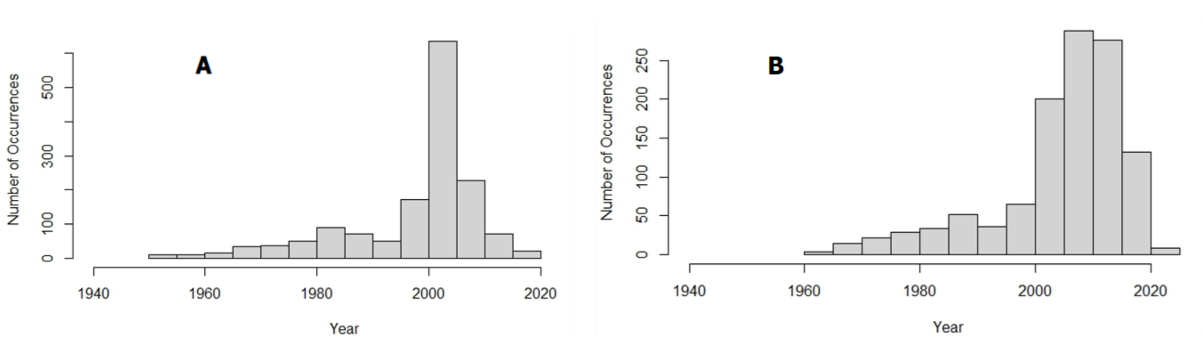

Supplementary Table S3: Top 10 vector model predictors

H. lusitanicum				*H. marginatum*			
RF		BRT		RF		BRT	
Predictor	Metric	Predictor	Metric	Predictor	Metric	Predictor	Metric
Rainfall Amplitude Component 1	20.44	Rainfall Amplitude Component 1	2.57	NDVI Minimum	9.84	Day Temperature Mean	1.66
Day Temperature Phase Component 2	17.2	Rainfall Amplitude Component 2	2.45	Rainfall Amplitude Component 2	9.8	NDVI Minimum	1.62
Rainfall Minimum	11.68	Day Temperature Phase Component 2	2.17	Day Temperature Mean	7.3	Rainfall Amplitude Component 2	1.55
Night Temperature Amplitude Component 1	7.1	Infra Red Amplitude Component 1	1.92	Rainfall Phase Component 3	4.36	Rainfall Amplitude Component 1	1.46
Night Temperature Minimum	4.12	EVI Phase Component 1	1.62	Rainfall Phase Component 1	4.34	Night Temperature Phase Component 2	1.41
Rainfall Amplitude Component 2	3.8	Infra Red Phase Component 1	1.52	Bare Ground Proportion	3.48	Night Temperature Maximum	1.35
Rainfall Phase Component 2	2.91	Night Temperature Amplitude Component 1	1.52	Night Temperature Phase Component 2	2.95	Rainfall Maximum	1.29
Infra Red Phase Component 1	2.59	Day Temperature Mean	1.4	Relative Humidity minimum	2.85	Day Temperature Phase Component 1	1.29
Night Temperature Mean	2.12	NDVI Phase Component 1	1.3	Relative Humidity Phase Component 3	2.8	NDVI Amplitude Component 2	1.26
Relative Humidity Amplitude Component 2	1.91	Infra Red Phase Component 2	1.28	Rainfall Phase Component 2	2.77	Day Temperature Maximum	1.25

Note: for both RF and BRT the higher the metric within each method the more important the predictor. The Metrics are different for each method, and should not be compared

Supplementary Figure S5: Full extent of modelled CCHF suitability map

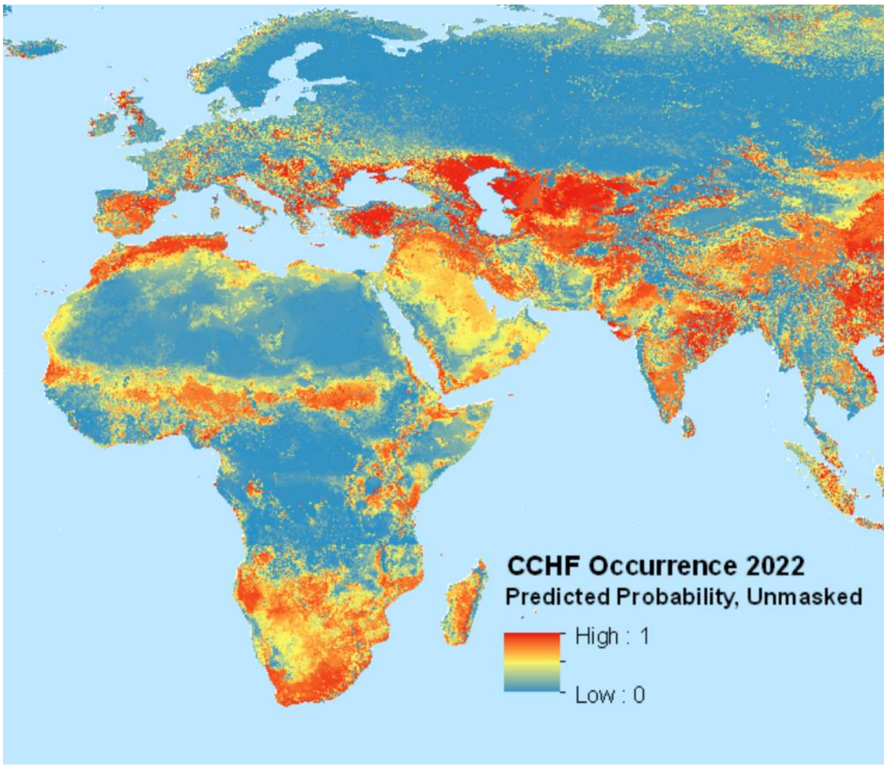

Supplementary Figure S6: Uncertainty estimates for CCHF suitability estimates (probability of occurrence)

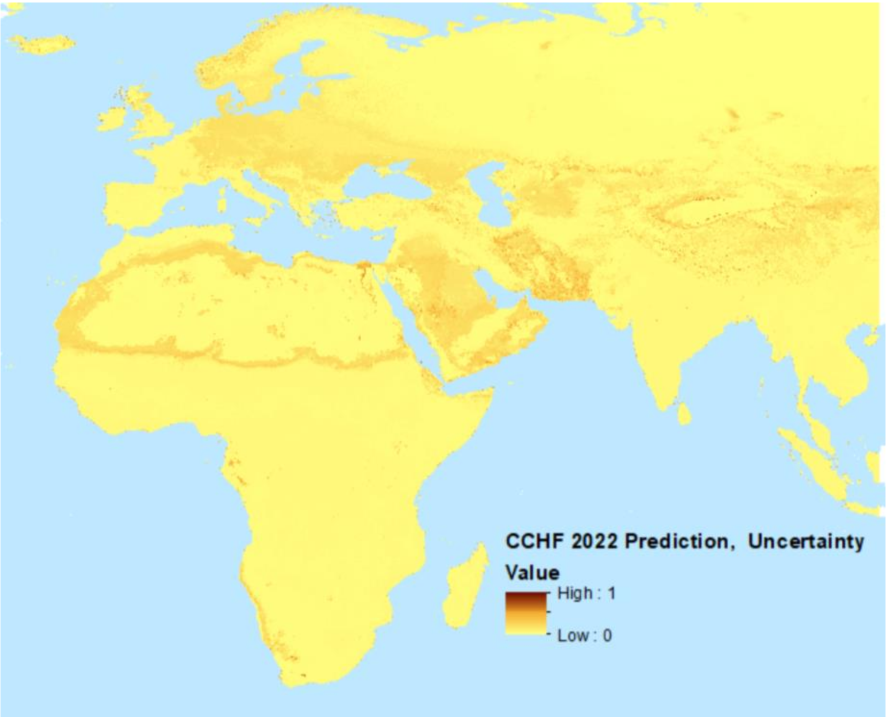

Uncertainty ranges from 0 to 1, with higher values indicating greater ranges in the estimated probability of occurrence.

Supplementary Figure S7: Unmasked 2015 CCHF prediction from Messina et al (2015).

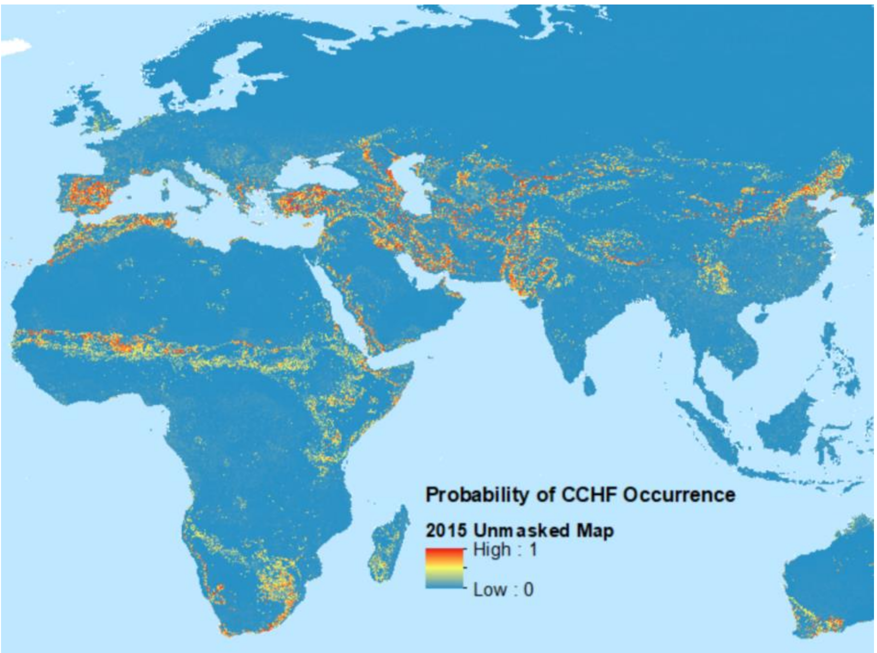

Supplementary Figure S8: Areas with > 0.75 probability of vector presence

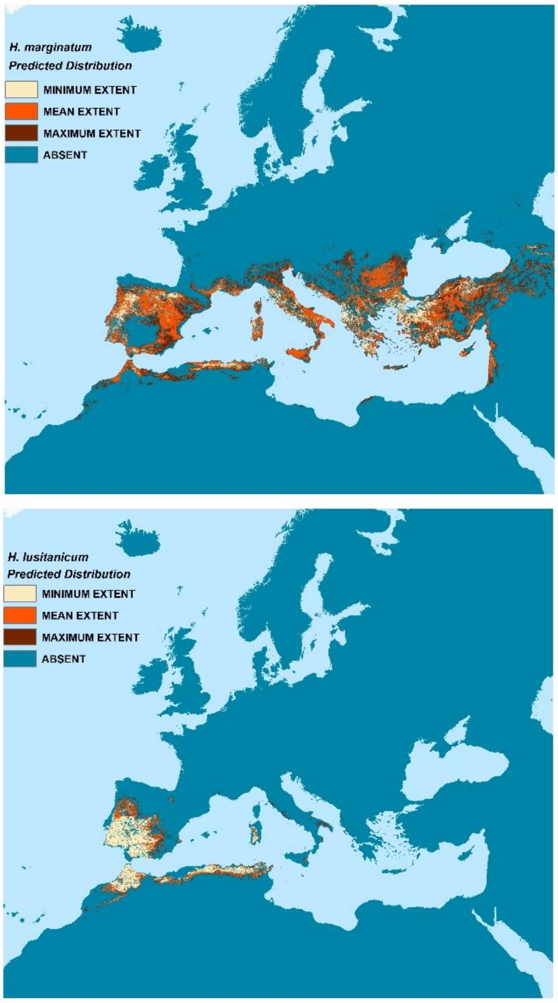

Supplement: Supplementary file 1 [file insects-14-00771-s001.zip › insects-2584735-supplementary.pdf]
